# Sex-dependent role of microglia in disulfide high mobility group box 1 protein-mediated mechanical hypersensitivity

**DOI:** 10.1097/j.pain.0000000000002033

**Published:** 2020-08-05

**Authors:** Nilesh M. Agalave, Resti Rudjito, Alex Bersellini Farinotti, Payam Emami Khoonsari, Katalin Sandor, Yuki Nomura, Thomas A. Szabo-Pardi, Carlos Morado Urbina, Vinko Palada, Theodore J. Price, Helena Erlandsson Harris, Michael D. Burton, Kim Kultima, Camilla I. Svensson

**Affiliations:** a Department of Physiology and Pharmacology, Center for Molecular Medicine, Karolinska Institutet, Stockholm, Sweden; b Department of Neuroscience, Neuroimmunology and Behavior Group, School of Behavioral and Brain Sciences, University of Texas at Dallas, Richardson, TX, United States; c Department of Medical Sciences, Clinical Chemistry, Uppsala University, Uppsala, Sweden; d Department of Neuroscience, Pain Neurobiology Research Group, School of Behavioral and Brain Sciences, University of Texas at Dallas, Richardson, TX, United States; e Department of Medicine, Center for Molecular Medicine, Karolinska Institutet, Stockholm, Sweden

**Keywords:** HMGB1, Microglia, Sex dimorphism, Spinal, LC-MS/MS, Minocycline, Pain

## Abstract

High mobility group box 1 protein (HMGB1) is increasingly regarded as an important player in the spinal regulation of chronic pain. Although it has been reported that HMGB1 induces spinal glial activation in a Toll-like receptor (TLR)4-dependent fashion, the aspect of sexual dimorphisms has not been thoroughly addressed. Here, we examined whether the action of TLR4-activating, partially reduced disulfide HMGB1 on microglia induces nociceptive behaviors in a sex-dependent manner. We found disulfide HMGB1 to equally increase microglial Iba1 immunoreactivity in lumbar spinal dorsal horn in male and female mice, but evoke higher cytokine and chemokine expression in primary microglial culture derived from males compared to females. Interestingly, TLR4 ablation in myeloid-derived cells, which include microglia, only protected male mice from developing HMGB1-induced mechanical hypersensitivity. Spinal administration of the glial inhibitor, minocycline, with disulfide HMGB1 also prevented pain-like behavior in male mice. To further explore sex difference, we examined the global spinal protein expression using liquid chromatography-mass spectrometry and found several antinociceptive and anti-inflammatory proteins to be upregulated in only male mice subjected to minocycline. One of the proteins elevated, alpha-1-antitrypsin, partially protected males but not females from developing HMGB1-induced pain. Targeting downstream proteins of alpha-1-antitrypsin failed to produce robust sex differences in pain-like behavior, suggesting that several proteins identified by liquid chromatography-mass spectrometry are required to modulate the effects. Taken together, the current study highlights the importance of mapping sex dimorphisms in pain mechanisms and point to processes potentially involved in the spinal antinociceptive effect of microglial inhibition in male mice.

## Abstract 2

Supplemental Digital Content is Available in the Text.

Disulfide high mobility group box 1 protein activates microglia and induces pain-like behavior in male and female mice, but the mechanisms underlying its central pronociceptive effects are sex-dependent.

## 1. Introduction

Although originally described as a nuclear protein, high mobility group box 1 protein (HMGB1) has reemerged as a pathogenic mediator of various diseases and injured states, including chronic pain.^4^ High mobility group box 1 protein can be passively or actively released by various cells, of which immune cells, glial cells, and neurons have been suggested as sources of nociception-modulating HMGB1.^4,23,49^ Conversely, endogenous HMGB1 induces production of inflammatory mediators that consequently stimulate glial cells and neurons, and thus nociception.^5,52^ The redox state of extracellular HMGB1 serves as a key regulator of receptor binding and immune responses. Reduction of cysteines at positions C23, C45, and C106 generates all-thiol HMGB1, which binds to receptor for advanced glycation end-products (RAGE) and evokes chemotactic activity through interactions with the C-X-C motif chemokine ligand 12 (CCXL12) and its receptor CXCR4.^59^ By contrast, disulfide HMGB1 is formed due to disulfide linkage between C23 and C45 and serves as a cytokine-inducing Toll-like receptor (TLR)4 ligand.^64^ Although initial studies on HMGB1 isoforms and receptor interactions have been exclusively conducted in male subjects, accumulating data show that similar interactions also occur in females.^1,3,5^

Spinal delivery of recombinant HMGB1 lowers the response threshold to calibrated touch and pressure stimuli in naive rats and mice.^3,45^ This action of HMGB1 has been coupled to TLR4 activation because only disulfide HMGB1 induces mechanical hypersensitivity after intrathecal (i.t.) injection, which is prevented in TLR4-deficient mice. In addition, spinal disulfide HMGB1 upregulates a myriad of factors frequently associated with glial activation in a TLR4-dependent fashion.^3^

Microglia are tissue-resident immune cells of the central nervous system, highlighted in recent years as having important roles in pain. In particular, there is a growing body of evidence that argues for a sexually dimorphic microglial involvement in chronic pain. Conventionally, most pain studies have been conducted in male rodents; however, accumulating evidence points to sex differences in pain mechanisms. Of note, it has been suggested that microglia are only required for pain processing in males, whereas females use the adaptive immune system. This proposition is based on observations that i.t. injection of microglial inhibitors reversed mechanical allodynia in males, whereas T-cell involvement was reported in females.^55^ Furthermore, others show that spinal TLR4 mediates inflammatory and neuropathic pain in male but not female mice.^54^ These findings support a sex-dependent role of microglia in nociception because TLR4 is known to regulate microglial activation.^35^ Nonetheless, the debate is still ongoing as others fail to report clear sex differences regarding the roles of both spinal microglia and TLR4.^17,62^ We have previously found that i.t. disulfide HMGB1 induces pain-like behavior in both male and female mice, and attenuating the action of HMGB1 alleviates mechanical hypersensitivity in both sexes subjected to arthritis.^3^ Thus, the purpose of this study was to examine whether disulfide HMGB1 induces nociceptive signaling through microglial activation in a sex-dependent fashion. We also undertook a global protein approach to uncover potential targets that may underlie sex differences in male and female mice.

## 2. Materials and methods

### 2.1. Animals

All experiments were conducted in accordance with protocols approved by the local ethics committee for animal experiments in Sweden (Stockholm North Animal Ethics Board) and the United States (the Institutional Animal Care and Use Committee of the University of Texas at Dallas), and were in accordance with the International Association for the Study of Pain guidelines. C57BL/6 male and female mice (10-12 weeks, 20-25 g) were purchased from Charles River Laboratories (Freiberg, Germany) and Janvier Labs (Le Genest-Saint-Isle, France). Myeloid cell-specific TLR4 depleted male and female mice (LysM-TLR4^fl/fl^) were generated as previously described.^25^ In brief, TLR^fl/fl^ mice were crossed with mice expressing Cre under LysM promoter. The resulting LysM-TLR4^fl/fl^ and TLR4^fl/fl^ (control mice) were backcrossed 8 generations to a C57BL/6 background at the University of Texas at Dallas. Wild-type (PAR2^+/+^) and proteinase-activated receptor 2 (PAR2)-deficient (PAR2^−/−^) male and female mice were obtained from Jackson Laboratory (Bar Harbor, ME) and bred at University of Texas at Dallas. Animals were housed in a pathogen-free environment with 5 mice per cage with water and food ad libitum in animal facilities at Karolinska Institutet and University of Texas at Dallas in a pathogen-free environment with standard temperature and 12-hour light/dark cycle.

### 2.2. Drugs and drug delivery

Endotoxin-free, disulfide HMGB1 was kindly provided by Dr. H. Yang (Feinstein Institute for Medical Research, NY) or purchased from HMGBiotech (Milan, Italy). Minocycline (glial inhibitor), alpha-1-antitrypsin (A1AT, protease inhibitor), haptoglobin (sequesters HMGB1) and sivelestat (neutrophil elastase inhibitor) were all purchased from Sigma-Aldrich (St. Louis, MO). Intrathecal (i.t.) delivery was performed in animals deeply anesthetized with 4% isoflurane and maintained on 2.5% isoflurane during the lumbar puncture procedure using a 30-gauge needle inserted into the space between L4-L5 vertebrae. Tail flick was noted as an indicator for a successful i.t injection. Animals were injected i.t with 1 μg disulfide HMGB1 or phosphate buffered saline (PBS) as vehicle control. In pharmacological experiments, 1 μg disulfide HMGB1 was injected i.t. either alone or in combination with 30 μg minocycline, 15 μg haptoglobin, 15 ng A1AT, or 0.5 ng sivelestat. The following day, the animals were given i.t. injection of either 30 μg minocycline, 30 ng A1AT, 1 ng sivelestat, or vehicle (PBS). All reagents were injected in a total volume of 5 μL i.t.

### 2.3. Microglial culture

The murine microglial cell line N13 (CD1 strain) was kindly provided by Prof. Gunnar Schulte, Karolinska Institutet, Stockholm, Sweden. Cells were expanded in Dulbecco's modified Eagle's medium (DMEM, Gibco, Carlsbad, CA) supplemented with 10% fetal bovine serum (FBS, Sigma-Aldrich), 2 mM L-glutamine, 50 U/mL penicillin, and 50 μg/mL streptomycin (Gibco) in a humidified incubator with 5% CO_2_. Upon reaching 75% to 80% confluency, cells were dissociated with 0.25% trypsin-ethylenediaminetetraacetic acid (trypsin-EDTA, Sigma Aldrich) and seeded at 2.5 × 10^5^ cells/well in 12-well plate in supplemented DMEM medium. After overnight incubation to allow for cell attachment, cells were serum starved (0.5% FBS) for 24 hours. Cells were stimulated with 1 μg/mL disulfide HMGB1 and collected 6 hours later for quantitative real-time polymerase chain reaction (qPCR) analysis.

To generate primary microglial culture, male and female mice were deeply anesthetized with isoflurane and intracardially perfused with ice-cold PBS containing 5 mM EDTA to reduce the presence of blood mononuclear cells. After decapitation, the skull cap was removed, and brain tissues were collected. Spinal column was cut at the level of the iliac crest, and the spinal cord was extracted using hydroextrusion with 21-gauge needle and ice-cold PBS. Both tissues were then transferred into a 15 mL conical tube containing ice-cold PBS until homogenization. To maximize cell yield, brain and spinal cord tissues from 2 to 3 mice were combined from the same sex for homogenization in each experiment. Microglia were then isolated and cultured using a modified version of previously described protocols.^2,53^ In brief, tissues were mechanically dissociated using sterile scalpels followed by enzymatic digestion with papain (2 mg/mL, Sigma-Aldrich) for 30 minutes at 37°C. The homogenate was centrifuged at 500*g* for 5 minutes at 4°C, and microglial cells were isolated using Percoll density gradient. First, cell pellets were resuspended in microglial culture medium containing DMEM/nutrient mixture F-12 (DME/F12; Gibco) supplemented with 10% FBS (Sigma-Aldrich), 100 U/mL penicillin, 50 μg/mL streptomycin, and 2 mM glutamine (Gibco), and centrifuged at 500*g* for 30 minutes at room temperature over a 37%/70% Percoll gradient (GE Healthcare, Princeton, NJ). Microglial cells were then collected from the interface, and the total cell number was determined using hemocytometer. Cells were seeded at 2.5 × 10^5^ cells/well in a 48-well plate precoated with poly-D-lysine (Sigma-Aldrich) and incubated for 2 hours at 37°C in microglial culture medium to allow for cell attachment. Subsequently, cells were serum starved (2% FBS) and maintained for 24 hours in a humidified 37°C incubator with 5% CO_2_. The next day, cells were stimulated with 1 μg/mL disulfide HMGB1 and collected 6 hours later for qPCR analysis.

### 2.4. Behavioral tests

Animals were placed in wire mesh bottomed cages and allowed to habituate to the test environment before baseline measurement. Three baselines were measured on 3 different days followed by randomization of the animals into treatment groups. Mechanical hypersensitivity was assessed using calibrated von Frey filaments (Marstock, Marburg, Germany; and Stoelting, Wood Dale, IL) applied on the plantar surface of the hind paws using the up-down method.^12^ The filament was applied for 2 to 3 seconds or until a brisk withdrawal was observed. Paw withdrawal thresholds were recorded in grams and calculated as percentage change of the respective mean baseline values. The effect of i.t. disulfide HMGB1 on mechanical hypersensitivity in wild-type and genetically modified mice (LysM-TLR4^fl/fl^ and PAR2^−/−^) was assessed 6 hours and then once a day until day 5 postinjection. In pharmacological experiments with minocycline, haptoglobin, alpha-1-antitrypsin, and sivelestat, mechanical hypersensitivity was evaluated 3, 6, and 24 hours after the first i.t. injection when HMGB1 and one of the inhibitors were coadministered, and then 3 and 6 hours after the second i.t. injection when the inhibitors were injected alone. All behavioral experiments were performed during day cycle in a blinded fashion.

### 2.5. Quantitative real-time PCR

Cells were washed with prechilled PBS and homogenized in TRIzol reagent (Invitrogen, Carlsbad, CA). mRNA was extracted according to manufacturer's protocol followed by cDNA synthesis using MultiScribe Reverse Transcriptase (Invitrogen) and qPCR using StepOne Real-Time PCR Systems (Applied Biosystems, Foster City, CA). Predeveloped probes for *Tnf* (Mm00443258_m1), *Il1b* (Mm00434228_m1), *Il6* (Mm00446190_m1), *Ccl2* (Mm00441242_m1), and *Hprt1* (Mm01545399_m1) (all from Applied Biosystems) were used for mRNA analyses. Threshold cycle values for each sample were used to calculate the number of cell equivalents using the standard curve method.^11^ The data were normalized to the housekeeping *Hprt1* mRNA levels and expressed as percentage change of the control group.

### 2.6. Immunohistochemistry

Animals were deeply anesthetized with isoflurane and intracardially perfused with PBS followed by 4% formaldehyde at 24 hours after i.t. injection of either 1 μg disulfide HMGB1 or PBS. Lumbar spinal cords (L3-L5) were then dissected followed by postfixation in 4% formaldehyde for 20 hours and cryoprotection in 20% sucrose solution for 24 hours. Spinal cords were embedded in optical cutting temperature compound (Histolab, Gothenburg, Sweden), frozen with liquid CO_2_, and cut at 20 μM using CryoStar NX70 cryostat (Thermo Fisher Scientific, Bremen, Germany). Tissue sections were incubated with rabbit anti-Iba1 antibody (1:2000, cat no. 019-19741, Wako, Richmond, VA) and horseradish peroxidase-conjugated anti-rabbit secondary antibody (1:200, cat no. P0448, Dako, Glostrup, Denmark). Immunoreactivity was visualized with tyramide signal amplification Plus kit (Perkin Elmer, Waltham, MA) according to manufacturer's protocol. Stained sections were mounted with Prolong Gold antifade mounting medium (Thermo Fisher Scientific) and examined by LSM710 confocal microscope (Carl Zeiss, Jena, Germany). The immunoreactivity (IR) intensity of the left and right dorsal horns was measured after manually outlining laminae I-VI and subtracting background signals using ImageJ (NIH, Bethesda, MD). Three L4 sections separated at least 50 μM from each other were analyzed per animal and the IR intensity averaged. An increase in IR intensity was considered as a sign of increased microglial reactivity.^47^ The analyses were performed by 2 independent investigators who were blinded to the treatment groups.

### 2.7. Protein extraction

Mice were euthanized 6 hours postinjection and lumbar spinal cords were collected by hydroextrusion, snap-frozen, and stored at −80°C. For protein extraction, samples were disrupted in a lysis buffer containing PBS, 1% sodium dodecyl sulphate (SDS), and a cocktail of protease inhibitors (Roche, Mannheim, Germany) and subjected to repeated freeze thawing and sonication for 10 to 15 seconds (0.3 on 0.7-second pulse) using a tip sonicator. Protein concentration was estimated using standard Pierce bicinchoninic acid (BCA) assay kit (Thermo Fisher Scientific) according to manufacturer's protocol. For liquid chromatography-mass spectrometry (LC-MS/MS), protein samples were further alkylated and reduced by the addition of dithiothreitol and iodoacetamide (Sigma-Aldrich), followed by overnight acetone precipitation. Protein pellets were then enzymatically digested in endoproteases Lys-C and trypsin (Sigma-Aldrich) overnight and stored at −20 °C.

### 2.8. Liquid chromatography-mass spectrometry

The peptide samples were labeled using tandem mass tag 6 (TMT6) plex isobaric label reagent set (Thermo Fisher Scientific) according to manufacturer's protocol. One mouse from each treatment group (4 in total) was assigned to a TMT6 plex, including 2 pools of all samples for each TMT6 plex. The TMT-labeled peptide samples were prefractionated using high pH reverse-phase liquid chromatography (12 fractions that were pooled into 5 fractions for each TMT6 plex). The fractions were evaporated, reconstituted in 20 µL 0.1% formic acid, and 4 µL were analyzed by high-resolution nano LC-MS/MS using an Q-Exactive mass spectrometer (Thermo Fisher Scientific) coupled to high-performance nanoLC systems (Dionex Ultimate-3000; Thermo Fisher Scientific) set up in a trap (Acclaim PepMap 2 cm, 3 μm C18) and elute (50 cm EasySpray PepMap RSLC C-18) configuration (both Thermo Fisher Scientific). The LC gradient buffers consisted of A (3% acetonitrile, 0.1% formic acid in LC-MS grade water) and B (96% acetonitrile, 0.1% formic acid in LC-MS grade water) components. The buffer B gradually increased from 3% to 37% in 150 minutes, to 47% in the next 20 minutes and 99% in the next 9 minutes. The column was equilibrated for an additional 11 minutes before the next sample was injected. Peptides were eluted at a flow rate of 250 nL/min and equilibration at 400 nL/min. MS spectra were acquired in a data-dependent acquisition (top 12) in full MS mode at 70,000 resolution (scan range of 300-1600 m/z) and further fragmented in MS/MS mode performed by collision-induced dissociation (arbitrary unit of 33) at 35,000 resolution (scan range of 100-2000 m/z).

### 2.9. Protein identification

The raw MS data were converted to an open-source format (mzML) by “msconvert” from ProteoWizard^30^ and processed using OpenMS^48^ through the following workflow: for identification, the UniProt/Swiss-Prot mus musculus database (release 2017_07) combined with a decoy database (the sequences were reversed) was used in MS-GF+ search engine^31^ (precursor mass tolerance: 10 ppm; enzyme: trypsin; min precursor_charge: 2; max precursor charge: 4; fixed modifications: Carbamidomethyl (C), TMT6plex (N-term and K); variable modifications: Oxidation (M), Deamidated (N and Q), Acetyl (N-term)). The result was imported into Percolator^27^ and peptides matches with a q-value <0.05 were used in Fido^50^ to score proteins based on peptide-spectrum matches. The proteins with q-values <0.05 were selected for subsequent analysis. For quantification, “IsobaricAnalyzer” was used to find and quantify the peptides using TMT6Plex correction matrix. The resulting features and peptides from the identification stages were mapped together using “IDMapper” (m/z tolerance: 5 ppm; RT tolerance: 2 seconds). The corresponding peptides and features across the samples were matched using the “FeatureLinkerUnlabeledQT” (m/z tolerance: 5; RT tolerance: 20 seconds). Peptide abundances were aggregated to protein abundances using “ProteinQuantifier,” in which the intensity of the peptides (protein q-values <0.05) were summed. The result was imported to the statistical software environment R (R Core Team (2013). R: A language and environment for statistical computing. R Foundation for Statistical Computing, Vienna, Austria. http://www.R-project.org/) and log_2_ transformed. The protein abundance in each sample was subtracted from the global pool within TMT set. The proteins were limited to the ones that were identified with at least 2 peptides and quantified in both treatment groups (irrespective of sex) with less than 50% missing values. Finally, the protein abundances were normalized using an in-house version of cyclic loess normalization^7^ (https://github.com/PayamEmami/limma).

### 2.10. Western blot

Proteins were separated using gel electrophoresis and transferred to nitrocellulose membrane (Thermo Fisher Scientific). Nonspecific binding sites were blocked with 5% nonfat milk in Tris based-buffer (50 mmol/L Tris-HCl and 6 mmol/L NaCl with 0.1% Tween 20). Then, the membranes were incubated overnight with primary antibodies followed by horseradish peroxidase-conjugated secondary antibodies. Primary antibodies used were goat anti-haptoglobin (1:1000, cat no. GHPT-90A-Z, ICL, Portland, OR), goat anti-alpha-1-antitrypsin (1:2000, cat no. AF2979, R&D Systems, Minneapolis, MN), sheep anti-ELA2 (1:1000, cat no. AF4517, R&D Systems), rabbit anti-PAR2 (1:250, cat no. 180953, Abcam, Cambridge, United Kingdom), and mouse anti-glyceraldehyde 3-phosphate dehydrogenase (1:10,000, cat no. ab8245, Abcam), whereas secondary antibodies used were anti-goat (1:7500, cat no. ab6685, Abcam), anti-sheep (1:5000, cat no. 12-342, Sigma-Aldrich), anti-rabbit (1:500, cat no. 7074, Cell Signaling, Danvers, MA), and anti-mouse (1:5000, cat no. 7076, Cell Signaling). Chemiluminescent reagent was used to visualize immunopositive bands (Thermo Fisher Scientific), and signal intensity was measured using Quantity One software (Bio-Rad, Hercules, CA). Immunopositive bands were normalized to the bands of glyceraldehyde 3-phosphate dehydrogenase, and results are presented as percentage change to the respective control group.

### 2.11. Statistical analyses

Differences between 2 groups were assessed by unpaired 2-tailed Student *t* test, whereas differences between groups split into 2 independent variables were analyzed by two-way analysis of variance followed by Tukey post hoc test using GraphPad Prism (San Diego, CA). *P*-values less than 0.05 were considered statistically significant. For LC-MS/MS data, the data were imported to *R*, transformed into log_2_ scale, and multiple linear models were fitted (using a classic 2 × 2 factorial design) through the proteins and moderated statistics was calculated (f-statistics for the global tests, t-statistics for individual contrasts and corresponding *P*-values and fold changes) using the “limma” package^46^ in *R*. The following 6 contrasts were included in the model: “Global sex difference: ((MalesH + MalesHM) − (FemalesH + FemalesHM)),” “Sex difference: (MalesH − FemaleH),” “Global treatment effect: ((MalesHM + FemalesHM) − (MalesH + FemalesH)),” “Treatment effect in males: (MalesHM − MalesH),” “Treatment effect in females: (FemalesHM − FemalesH),” “Difference in sex after treatment: (MalesHM − FemalesHM),” and “The interaction effect: (MalesHM − MalesH)—(FemalesHM-FemalesH).” “H” denotes only HMGB1 injection and “HM” denotes HMGB1 injection followed by minocycline. The moderated statistics were converted to regular statistics using an in-house function (https://github.com/PayamEmami/limma). To correct for multiple testing, the resulting *P*-values (analysis of variance *P*-values) were transformed to q-values^28^ using “q-value” package in *R*. For significant proteins (q-value <0.05), a Venn diagram was constructed showing the result of “Global sex difference,” “Global treatment effect,” and “The interaction effect,” using a *P*-value <0.05 for each individual contrast. To ease interpretation, bar plots using FemalesH as baseline (“MalesH − FemalesH,” “MalesHM − FemalesH,” and “FemalesHM − Females H”) were constructed.

## 3. Results

### 3.1. Intrathecal injection of disulfide high mobility group box 1 protein induces morphological changes related to microglial reactivity in lumbar spinal cords of male and female mice

**Figure 1.**
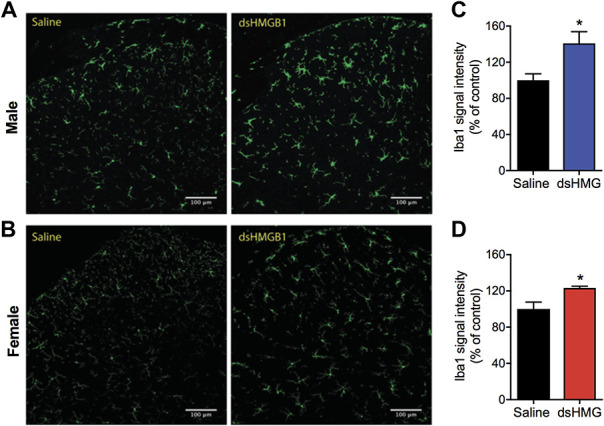
Intrathecal injection of disulfide HMGB1 leads to upregulation of Iba1 expression in spinal lumbar dorsal horn. Representative confocal images of Iba1 immunoreactivity in (A) male and (B) female spinal dorsal horns 24 hours postinjection of either vehicle (saline) or disulfide HMGB1. Bar graphs display quantification of Iba1 signal intensity in (C) male and (D) female mice expressed as percentage change to the control saline group. Data are presented as mean ± SEM, n = 5 to 6 mice/group. *P < 0.05 vs control group. dsHMG, disulfide HMGB1, scale bar: 100 μm. HMGB1, high mobility group box 1 protein.

In an earlier study, we have demonstrated that i.t. disulfide HMGB1 increases mRNA levels of the microglial marker *Cd11b* in the spinal cord. However, that study was only performed in male mice.^3^ Therefore, to examine whether male and female mice exhibit a similar pattern of microglial reactivity, we stained lumbar spinal cord sections with the microglial marker ionized calcium binding adaptor molecule 1 (Iba1). A morphological change in the form of larger cell bodies and more ramified processes was observed for the majority of Iba1-stained cells in the dorsal horn 24 hours after i.t. disulfide HMGB1 in both sexes (Figs. 1A and B). We observed significantly higher intensity of Iba1 signal in both male (140.9 ± 13.0 vs 100.0 ± 7.2%, n = 6, *P* = 0.02) and female (123.2 ± 1.9 vs 100.0 ± 7.6%, n = 5, *P* = 0.02) mice subjected to disulfide HMGB1 injection compared to the respective saline control groups (Figs. 1C and D).

### 3.2. Disulfide high mobility group box 1 protein stimulation induces higher proinflammatory mediators in cultured microglia derived from male compared to female mice

**Figure 2.**
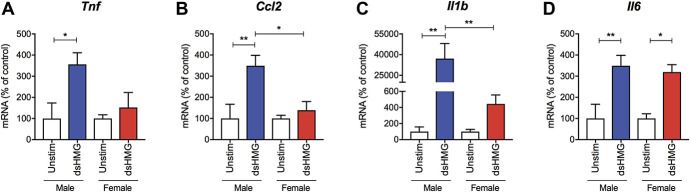
Disulfide HMGB1 induces cytokine and chemokine expression to higher levels in primary microglial culture generated from male compared to female mice. Bar graphs represent mRNA levels for (A) *Tnf*, (B) *Ccl2*, (C) *Il1b*, and (D) *Il6* in primary cultures of microglia 6 hours after stimulation with disulfide HMGB1 (1 μg/mL) or no stimulation. Data are represented as mean ± SEM, n = 4 to 5 experimental replicates/group, *P < 0.05, **P < 0.01 vs unstimulated group. Unstim, unstimulated; dsHMG, disulfide HMGB1; HMGB1, high mobility group box 1 protein.

We have previously shown that i.t. disulfide HMGB1 drives cytokine and chemokine expression frequently associated with glial activation in the spinal cord such as *Tnf*, *Ilb*, *Il6*, and *Ccl2*.^3^ To examine whether HMGB1 has a direct action on microglia, we first used the microglial cell line N13 and found that disulfide HMGB1 evoked increased gene expression of *Tnf*, *Ilb*, *Il6*, and *Ccl2* (Figs. S1A–D, available as supplemental digital content at http://links.lww.com/PAIN/B144). However, because the sex of the mouse used for establishment of the N13 cell line is not readily available, we used primary microglial culture generated from brain and spinal cord tissues to examine whether the response to disulfide HMGB1 differs between microglia from males vs females. Cultured microglia were stimulated with disulfide HMGB1 for 6 hours followed by mRNA isolation and measurement of cytokine and chemokine expression by qPCR. Exposure to disulfide HMGB1 for 6 hours led to an increase of *Tnf*, *Ccl2*, and *Il1b* mRNA levels compared to unstimulated cells in male-derived but not female-derived microglia, although there was a trend towards an increase of *Il1b* mRNA levels in female microglia after disulfide HMGB1 stimulation albeit not significant (Figs. 2A–C). By contrast, disulfide HMGB1 induced *Il6* expression to similar levels in both sexes (Fig. 2D). Because 2 to 3 mice of each sex were pooled to generate primary cultures in this experiment, n represents experimental replicates. The experiment was repeated twice with similar results.

### 3.3. Inhibition of spinal microglial function attenuates disulfide high mobility group box 1 protein-induced mechanical hypersensitivity in male but not female mice

**Figure 3.**
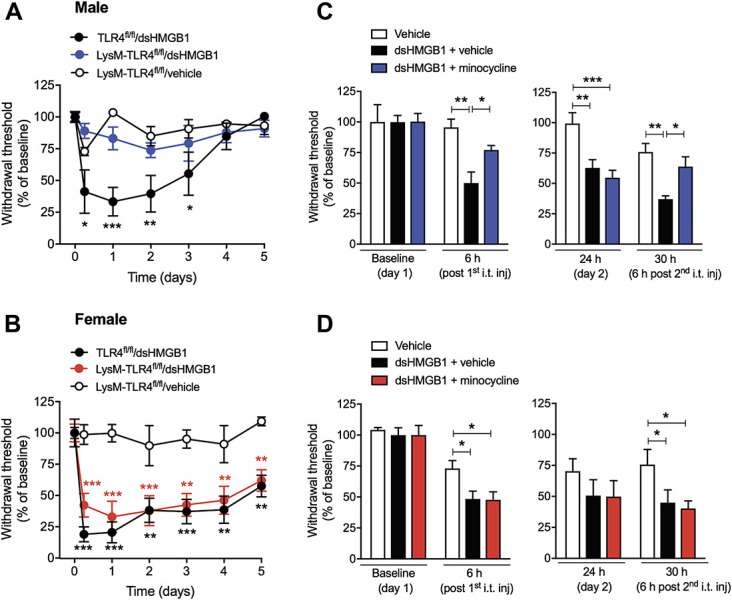
Blockade of microglial function elicits antinociceptive effects in disulfide HMGB1-subjected male but not female mice. Withdrawal threshold values after i.t. injection of disulfide HMGB1 (1 μg/mouse) or vehicle (saline) in (A) male and (B) female mice lacking TLR4 in microglia (LysM-TLR4^fl/fl^) or TLR4^fl/fl^ (control mice). Withdrawal response before, and 6 and 24 hours after the first day i.t. injection of a combination of disulfide HMGB1 (1 μg/mouse) and minocycline (30 μg/mouse, microglial inhibitor) or disulfide HMGB1 (1 μg/mouse) and vehicle (PBS), as well as 6 hours after the second day intrathecal injection of minocycline (30 μg/mouse) or vehicle in (C) male and (D) female mice. Data are presented as mean ± SEM, n = 4 to 8 mice/group, *P < 0.05, **P < 0.01, ***P < 0.001 vs vehicle groups. ds HMGB1, disulfide high mobility group box 1 protein.

Spinal injection of disulfide HMGB1 evokes mechanical hypersensitivity in both male and female mice^3^; however, whether the underlying mechanism is regulated by microglia in a sex-dependent fashion is not known yet. To investigate this, we undertook 2 different approaches to inhibit microglial function, and therefore disrupt their interaction with disulfide HMGB1 in both male and female mice. The first approach was using conditional knockout mice that lack TLR4 in myeloid-derived cells, which includes microglia (LysM-TLR4^fl/fl^).^25^ Spinal delivery of disulfide HMGB1 in LysM-TLR4^fl/fl^ mice did not elicit mechanical hypersensitivity in males (Fig. 3A), whereas females displayed lower mechanical thresholds than vehicle-injected mice and were of similar levels to the injected control TLR4^fl/fl^ mice (Fig. 3B). The second approach was using minocycline, a drug that is frequently used as a microglial inhibitor. Disulfide HMGB1 was injected i.t. either alone or in combination with minocycline, followed by a second i.t. injection of either vehicle (PBS) or minocycline 24 hours later. Coinjection with minocycline prevented disulfide HMGB1-induced mechanical hypersensitivity in male but not female mice 6 hours postinjection on the first day. A second minocycline injection the following day significantly reversed pain-like behavior only in male mice previously injected with disulfide HMGB1 (Figs. 3C and D).

### 3.4. Global protein expression profile of the lumbar spinal cords displays sex dimorphism in response to minocycline treatment

**Figure 4.**
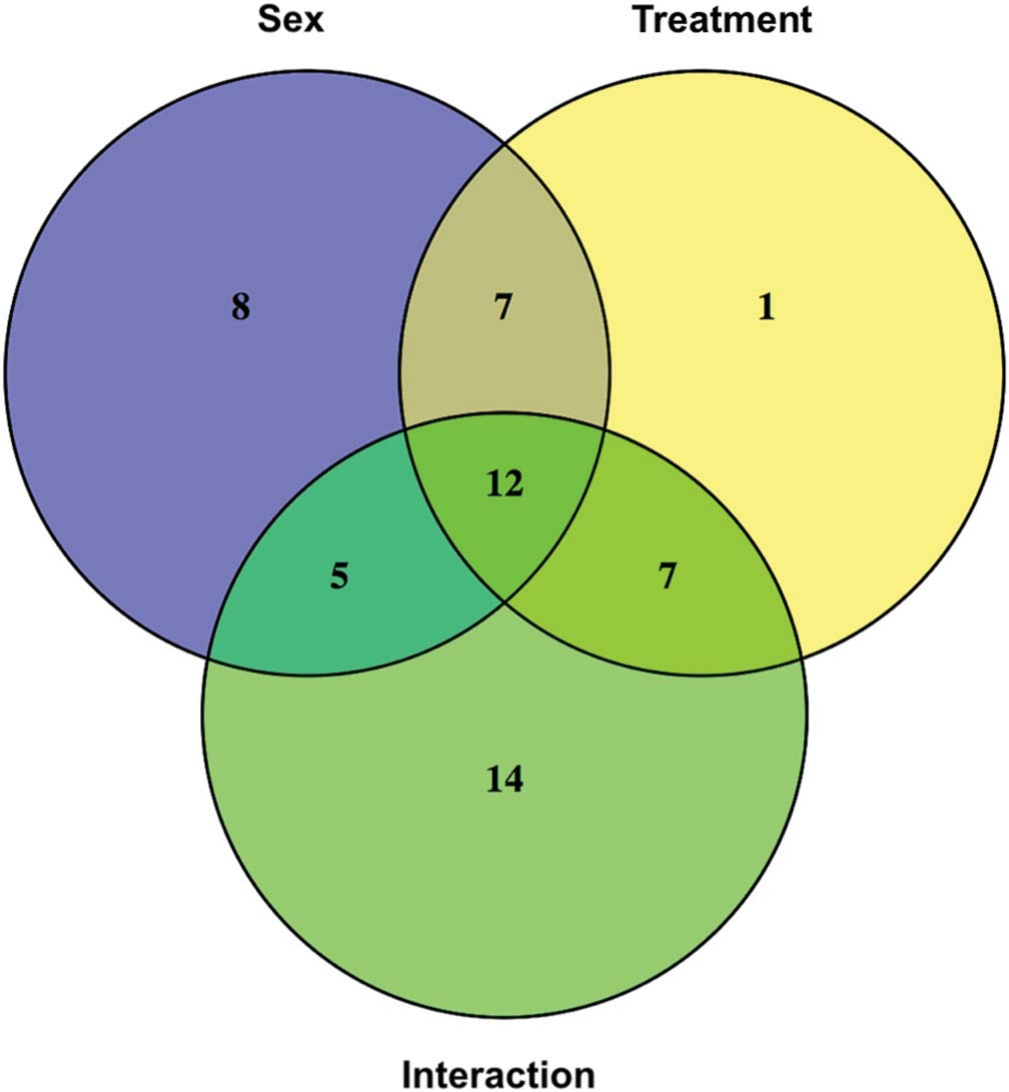
LC/MS-MS reveals 12 spinal proteins to be differentially expressed in response to sex and minocycline treatment. Venn diagram displays 3 statistically significant contrasts (*P* < 0.05)—sex difference, treatment effect and the interaction between sex and treatment—analyzed in the 54 proteins identified by LC-MS/MS. LC-MS/MS, liquid chromatography-mass spectrometry.

**Figure 5.**
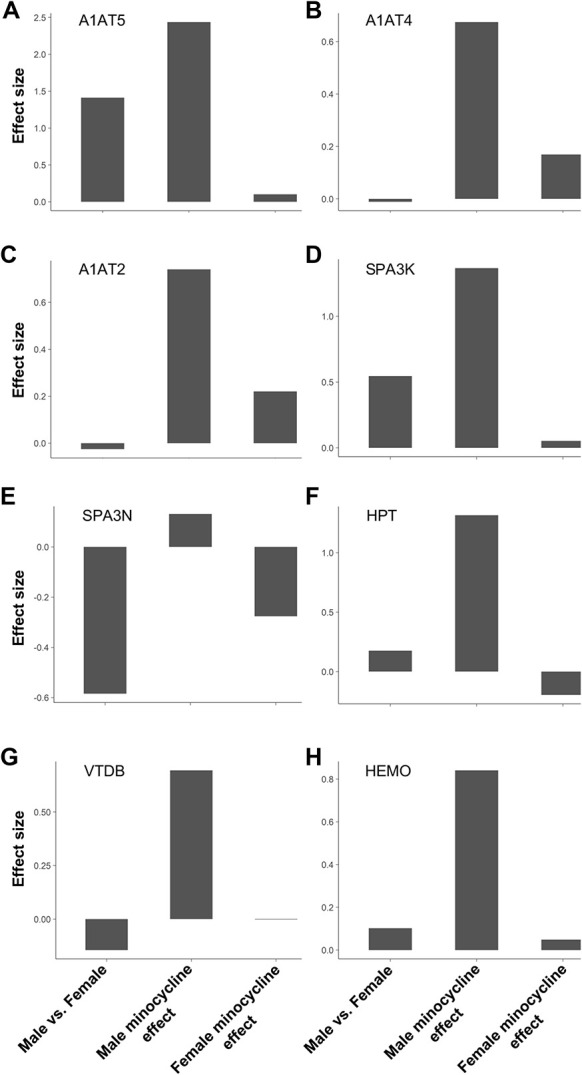
Minocycline treatment induces upregulation of anti-inflammatory and antinociceptive factors in the spinal dorsal horns of disulfide HMGB1-subjected male but not female mice. Bar graphs depict the effect size in protein expression of (A) A1AT5, (B) A1AT4, (C) A1AT2, (D) SPA3K, (E) SPA3N, (F) HPT, (G) VTDB, and (H) HEMO between (1) male vs female injected with disulfide HMGB1, (2) males injected with disulfide HMGB1-minocycline compared to disulfide HMGB1-vehicle, and (3) females injected with disulfide HMGB1-minocyline compared to disulfide HMGB1-vehicle. Data are presented as relative levels in log_2_ scale, n = 6 mice/group. HMGB1, high mobility group box 1 protein.

We used LC-MS/MS to identify possible targets that underlie sex differences in the spinal cords between male and female mice subjected to disulfide HMGB1 alone, or in combination with minocycline. The first treatment group is referred to as “vehicle” group, and the latter as “minocycline” group. Lumbar spinal cords were collected 6 hours after the second i.t. injection and subsequently processed for LC-MS/MS. In total, 2947 proteins were identified and relatively quantified. Using a 2 × 2 factorial design, we found 54 proteins to be differentially expressed in male and female mice in both vehicle and minocycline groups (q < 0.05). Out of these, 36 proteins were differentially expressed in both sexes in the vehicle group. In males, differences in expression were observed in 44 proteins between vehicle and minocycline group, which is in contrast to only 8 proteins being affected in females (Table S1 and Table S2, available as supplemental digital content at http://links.lww.com/PAIN/B144). Proteins that showed significant interaction between sexes and minocycline were narrowed down to 12 proteins (Fig. 4). A total of 8 out of 12 proteins were upregulated in male minocycline group as compared to vehicle group, whereas we observed minimal or opposite effects on female mice (Figs. 5A–H). The protein with the largest effect size between male and female vehicle groups was alpha-1-antitrypsin 1 to 5 (A1AT5). Interestingly, A1AT5 also showed the largest effect size in male mice in response to minocycline treatment (Fig. 5A). Besides A1AT5, other isoforms of alpha-1-antitrypsin such as A1AT4 and A1AT2 were upregulated in male minocycline group (Figs. 5B and C) alongside serine protease inhibitor A3K (SPA3K), SPA3N, haptoglobin (HPT), hemopexin (HEMO), and vitamin D binding protein (VTDB) (Figs. 5D–H). None of these proteins were significantly altered in response to the minocycline treatment in female mice (Figs. 5A–H).

### 3.5. Alpha-1-antitrypsin but not its downstream target neutrophil elastase elicits partial antinociceptive properties in a sex-dependent manner

By Western blot, we validated the changes in haptoglobin expression observed in LC/MS-MS in which haptoglobin was upregulated in male but downregulated in female mice in the minocycline group (Figs. S2A and B, available as supplemental digital content at http://links.lww.com/PAIN/B144). Recent work shows that haptoglobin binds to HMGB1 and counterregulates the proinflammatory properties of HMGB1 by skewing macrophage polarization towards an anti-inflammatory phenotype.^65^ Interestingly, this study was only conducted in male mice and therefore it is not known whether a similar action of haptoglobin on HMGB1 occurs in female mice. Given the fact that haptoglobin is elevated in male but reduced in female minocycline group suggests that haptoglobin may serve as a protective mechanism for HMGB1-induced hypersensitivity in male mice. Therefore, we examined whether haptoglobin may be responsible for the antinociceptive effect of minocycline in male mice. Our findings, however, show that coinjection of haptoglobin (15 μg) with disulfide HMGB1 (1 μg) did not prevent HMGB1-induced mechanical hypersensitivity in both male and female mice (Figs. S2C and D, available as supplemental digital content at http://links.lww.com/PAIN/B144).

**Figure 6.**
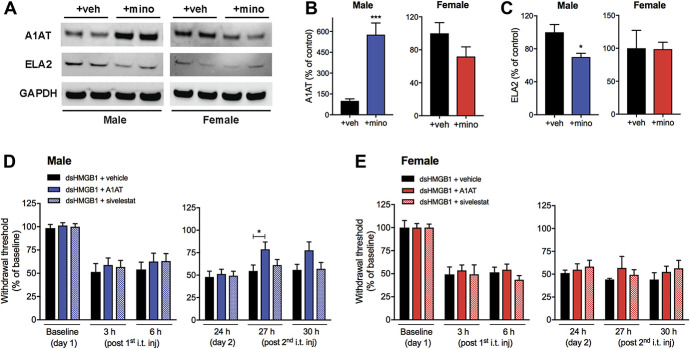
Alpha-1-antitrypsin, but not its downstream target neutrophil elastase, partially reverses disulfide HMGB1-induced pain-like behavior in male but not female mice. Representative Western blot images of (A) alpha-1-antitrypsin (A1AT), neutrophil elastase (ELA2), and glyceraldehyde 3-phosphate dehydrogenase (GAPDH) from protein extracts of lumbar spinal cords of male and female mice injected with disulfide HMGB1 in combination with vehicle (+veh) or minocycline (+mino). Bar graphs depict quantification of (B) A1AT and (C) ELA2 immunopositive bands normalized to GAPDH and presented as percentage change to vehicle control groups. Withdrawal response before, and 3, 6, and 24 hours after the first day i.t. injection of disulfide HMGB1 (1 μg/mouse) in combination with either alpha-1-antitrypsin (15 ng/mouse), sivelestat (0.5 ng, neutrophil elastase inhibitor), or vehicle (PBS), and 3 and 6 hours after the second day i.t. injection of either alpha-1-antitrypsin (30 ng/mouse), sivelestat (1 ng/mouse), or vehicle in (D) male and (E) female mice. Data are presented as mean ± SEM, n = 6 mice/group for Western blot results and n = 5 to 12 mice/group for behavioral results, *P < 0.05, ***P < 0.001 vs control groups. ds HMGB1, disulfide high mobility group box 1 protein.

The next target protein that we examined was alpha-1-antitrypsin and neutrophil elastase (Fig. 6A). Unlike humans, where only one essential gene is known, alpha-1-antitrypsin in mice occurs in multiple isoforms encoded by up to 5 polymorphic genes depending on the substrains.^9^ The strain that was used for this study, C57BL/6, harbors 5 paralogs, in which 3 out of 5 paralogs were upregulated in male mice of the minocycline group. We confirmed that alpha-1-antitrypsin expression was elevated in male but not female mice in response to minocycline treatment (male: 576 ± 84 vs 100 ± 15%, n = 6, *P* = 0.0002, female: 72 ± 12 vs 100 ± 13%, n = 6, *P* = 0.1) (Fig. 6B) by Western blot. Of note, the antibody used to detect alpha-1-antitrypsin does not differentiate between paralogs. Alpha-1-antitrypsin is characterized as a potent inhibitor of serine proteases, in particular neutrophil elastase,^9^ which has recently been implicated in both inflammatory and neuropathic pain.^43,44,60^ To examine whether elastase was a possible downstream target of the pronociceptive cascade inhibited by alpha-1-antitrypsin in male mice, we analyzed neutrophil elastase protein expression using Western blot. Indeed, we found a reduction of elastase (ELA2) protein expression in male mice treated with minocycline compared to vehicle (70 ± 4 vs 100 ± 9%, n = 6, *P* = 0.02) (Fig. 6C). By contrast, female mice showed equivalent levels of ELA2 expression in both minocycline and vehicle groups (99 ± 10 vs 100 ± 26%, n = 6, *P* = 0.9) (Fig. 6C).

Next, we investigated whether spinal injection of alpha-1-antitrypsin with disulfide HMGB1 would provide protection in mice from developing mechanical hypersensitivity. Coadministration of alpha-1-antitrypsin (15 ng/mouse) and disulfide HMGB1 (1 μg) into the spinal cord did not prevent HMGB1-induced pain-like behavior in either male or female mice (Figs. 6D and E). The following day, a higher dose of only alpha-1-antitrypsin (30 ng/mouse) was i.t. injected. A partial reversal in mechanical hypersensitivity was observed 3 hours after i.t. injection of alpha-1-antitrypsin in male, but not female mice (Figs. 6D and E). Interestingly, i.t. injection of the neutrophil elastase inhibitor sivelestat^29^ (0.5 ng) together with HMGB1 (1 μg) did not prevent or reverse disulfide HMGB1-induced pain-like behavior in either sex of mice (Figs. 6D and E), although the dose of sivelestat was doubled on the second day similar to the study with alpha-1-antitrypsin.

### 3.6. Proteinase-activated receptor 2 depletion partially protects both male and female mice from disulfide high mobility group box 1 protein-induced pain

**Figure 7.**
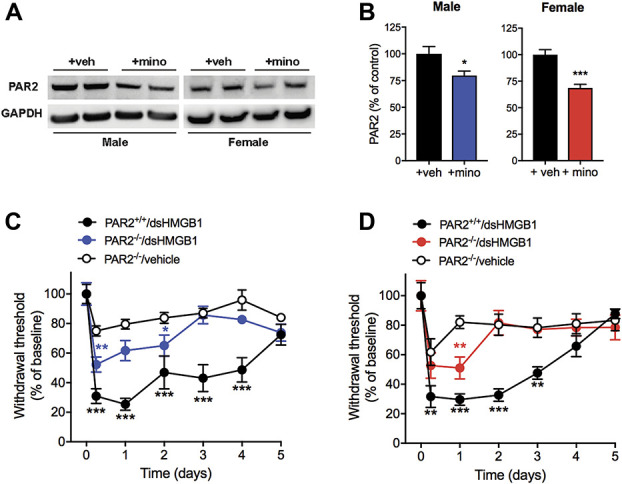
proteinase-activated receptor 2 depletion partially protects male and female mice from developing disulfide HMGB1-induced mechanical hypersensitivity. (A) Representative Western blot images and (B) quantification of proteinase-activated receptor 2 (PAR2) immunopositive bands normalized to glyceraldehyde 3-phosphate dehydrogenase (GAPDH) and presented as percentage change to vehicle control groups. Withdrawal response after i.t. injection of disulfide HMGB1 (1 μg) or vehicle in (C) male and (D) female wild-type (PAR2^+/+^) and whole-body PAR2 knockout (PAR2^−/−^) mice. Data are presented as mean ± SEM, n = 6 mice/group for Western blot results and n = 4 to 7 mice/group for behavioral results, *P < 0.05, **P < 0.01, ***P < 0.001 vs control groups. ds HMGB1, disulfide high mobility group box 1 protein.

Neutrophil elastase induces neurogenic inflammation and pain through activation of PAR2^44,67^ expressed by nociceptors.^22^ We hypothesized that alpha-1-antitrypsin directly inhibits neutrophil elastase, which subsequently blocks PAR2 activation. Counterintuitive to the results above, we found spinal PAR2 expression to be significantly decreased in both male and female mice receiving minocycline together with HMGB1 compared to vehicle i.t. (male: 80 ± 4 vs 100 ± 7%, n = 5, *P* = 0.03, female: 69 ± 6 vs 100 ± 5%, n = 6, *P* = 0.0003) (Figs. 7A and B). Of note, one data point in the male vehicle group was identified as an outlier using ROUT outlier test (Q = 1%) and therefore eliminated from the analysis. In accordance, both male and female whole-body PAR2 knockout (PAR2^−/−^) mice were partially protected from development of disulfide HMGB1-induced mechanical hypersensitivity (Figs. 7C and D).

## 4. Discussion

The current study investigates whether there is a sex-associated difference in TLR4-activating disulfide HMGB1-induced microglial activation and the mechanisms by which it drives pain-like behavior. Our results show that i.t. injection of disulfide HMGB1 increased microglial reactivity in both sexes based on morphology, but direct stimulation of cultured microglia with HMGB1 led to higher expression of cytokines and chemokines in males compared to females. Interestingly, attenuation of disulfide HMGB1-induced pain-like behavior was only achieved by the microglia inhibitor minocycline and TLR4 depletion in myeloid cells in male mice. We performed global proteomic analysis of the spinal cords, which revealed an increase in proteins associated with antinociceptive and anti-inflammatory effects in male receiving minocycline. Targeting these proteins alone, however, did not replicate the sex-dependent effects of minocycline on disulfide HMGB1-induced hypersensitivity. Together, these results suggest that microglia regulate HMGB1-induced pain mechanisms in a sex-dependent manner.

We have previously reported that i.t. injection of disulfide HMGB1 induces pain-like behavior in male and female mice^3^; however, it is not known whether disulfide HMGB1 promotes microglial activation similarly in both sexes. After i.t. injection of disulfide HMGB1, spinal microglia in both male and female mice display the characteristic signs of activation such as enlarged cell bodies and increased Iba1 immunoreactivity.^38,41,58^ This is supportive of previous reports showing similar levels of microglial activation in vivo after nerve injury.^54,56^ Another feature of activated microglia is cellular production of proinflammatory mediators.^10,57^ In agreement with previous work showing that TLR4 activation by lipopolysaccharide induces higher *Il1b* mRNA in male microglia,^37^ we found that disulfide HMGB1 stimulation of cultured primary microglia induces more pronounced mRNA levels of *Il1b* and other cytokines in male-derived cells compared to the females. Thus, the degree of proinflammatory response of microglia to not only lipopolysaccharide but also HMGB1 stimulation is sex-dependent.

We found that blockade of microglial activation by disulfide HMGB1 resulted in prevention of pain-like behavior only in male mice. Our findings support earlier reports showing that spinal microglia and TLR4 are important for nociception in males.^54,55^ Given the fact that disulfide HMGB1 induced higher mRNA levels of inflammatory factors in male compared to female microglia supports the notion that there is a greater microglial involvement in male mice. High mobility group box 1 protein can increase microglial activity through TLR2, TLR4, and RAGE receptor activation.^18^ When it comes to the redox form of HMGB1, it is not known how long HMGB1 is retained in the disulfide form in the spinal cord after i.t. injection and if it differs between male and female mice. Thus, it is possible that the redox state of HMGB1 change over time in a fashion that enables HMGB1 to start to act on other receptors such as TLR2,^6^ TLR5,^15^ RAGE,^32^ or CD24/siglec10.^63^ If this modification is differentially regulated between male and female mice, it is possible that another receptor system mediates the effect of i.t. HMGB1 in female mice, hence interfering with HMGB1-TLR4 interactions in females do not prevent pain-related behaviors.

To generate mice with TLR4-specific deletion in microglia, we used LysM-Cre mice, which is important to note because LysM activity is present in cells within the myeloid lineage such as macrophages.^25^ Several reports including our own^17^ have shown that microglia are the only resident myeloid cells present in the spinal cord parenchyma in naive states,^19,51^ therefore the loss of TLR4 activity in LysM-TLR4^fl/fl^ mice in the spinal cord is predominantly in microglia. It has been reported that LysM-Cre target only a subset of microglia (20%-45% out of the total population^20,61^), and it is noteworthy that targeting this subpopulation alone was sufficient to uncover the sex differences in our study.

Minocycline is frequently used to reduce microglial activity and here we show that minocycline only attenuated pain-like behavior in male mice. In agreement with our work, i.t. administration of this drug attenuates both neuropathic and inflammatory pain-like behavior in male rodents.^17,24,34,36,40,55^ Furthermore, i.t. minocycline has been shown to be ineffective in reducing pain-like behavior in female rodents evoked by nerve injury,^55^ intraplantar formalin injection,^13^ and antibody-induced arthritis,^17^ but was antinociceptive in bone cancer-induced pain model.^66^ These studies use different models, species, or strains of rodents, doses of minocycline, and timing of administration, which may account for different outcomes of minocycline treatment on pain-like behavior. A thorough investigation is thus needed to understand the inconsistencies of the antinociceptive effects of minocycline in females. Moreover, it is often oversimplified when relying on minocycline as a microglia inhibitor because this drug has well-documented anti-inflammatory properties that are not limited to microglia. Indeed, it is reducing microglial activity, but it is important to take into consideration that minocycline also can act on other cells, including T cells, neutrophils, astrocytes, and neurons.^42^ Still, the fact that minocycline has sex-associated antinociceptive effects in some animal models of pain testifies that there are differences between male and female pain mechanisms, and these should be further dissected.

As the sex-dependent effect of minocycline has been confirmed by several studies, we used in-depth proteomics with LC/MS-MS to further examine the downstream proteins regulating these sex differences. Our study highlights an interesting pattern that there is an upregulation of anti-inflammatory and antinociceptive proteins in male but not female mice that received minocycline. In particular, proteins of the serpin family, which are inhibitors of serine proteases, were well represented with levels of 5 serpin proteases, A1AT2, A1AT4, A1AT5, SPA3K, and SPA3N, being significantly elevated in spinal cords from minocycline-treated male mice. On the contrary, we were surprised to find only few proteins being downregulated in response to minocycline. This could be a matter of such factors as (1) being below detection limit, (2) being diluted during sample preparation as we here took a general approach rather than for example focus on membrane proteins, (3) being regulated by posttranslational modifications, and (4) having a lipid-based composition. In addition, changes in protein expression levels could arise as primary or secondary consequences of minocycline treatment and therefore our study does not delineate the cellular specificity of minocycline.

Targeting individual proteins that were upregulated after minocycline treatment produced none or modest antinociception. First, coadministration of haptoglobin with disulfide HMGB1 did not prevent pain-like behavior. As 3 out of 5 paralogs of alpha-1-antitrypsin were upregulated in the LC-MS/MS data, we assessed the ability of alpha-1-antitrypsin to prevent and reverse i.t. disulfide HMGB1-induced hypersensitivity. Interestingly, alpha-1-antitrypsin partially reversed disulfide HMGB1-induced pain-like behavior in male but not female mice. Recent reports show that peripheral administration of alpha-1-antitrypsin is antinociceptive by preventing activation of neutrophil elastase and PAR2.^43,44^ However, our results do not entirely support this linkage because we failed to demonstrate that inhibition of neutrophil elastase prevents disulfide HMGB1-induced hypersensitivity. In addition to neutrophil elastase, alpha-1-antitrypsin also blocks cathepsin G and proteinase 3.^39^ Hence, other proteases may be the link between alpha-1-antitrypsin and PAR2 activation, and the reason for why the antagonist sivelestat was not effective in alleviating hypersensitivity induced by disulfide HMGB1.

Although spinal PAR2 has been implicated in several pain models,^8,14,21,26,43,44,67^ our study is the first to consider sex differences. Interestingly, PAR2 expression was downregulated in both sexes after minocycline treatment and PAR2 depletion ameliorated disulfide HMGB1-induced pain in both male and female mice. In the light of our hypothesis regarding the sex-dependent effect of minocycline, this finding was rather unexpected. PAR2 is not only activated by neutrophil elastase, but also by trypsin and tryptase, which have been detected in the central nervous system.^16,33^ Thus, it is possible that disulfide HMGB1 induces different signaling pathways in males and females, which converge in PAR2 activation, and that minocycline only affects the linkage in male mice. Pinpointing the downstream signaling pathway that governs the sexual dimorphic effect of minocycline on HMGB1-induced pain seems to be complex. Therefore, further investigations of this as well as the other proteins identified by LC-MS/MS are warranted to gain a better understanding of how these proteins interact and modulate the sex-dependent effects of minocycline.

In conclusion, although disulfide HMGB1 induces pain-like behavior in both male and female mice, the mechanisms underlying its central pronociceptive effects on microglia are sex-dependent. Our findings support previous works showing that sexually dimorphic pain processing is present not only in humans but also in rodents, and highlights the importance of including animals of both sexes to optimize the translational potential of preclinical pain research. Our global protein approach uncovers an intriguing pattern suggesting differences in microglial function between males and females in which minocycline elevates factors associated with anti-inflammatory and antinociceptive properties in male mice only, but that there may be points of convergence. Several of the proteins identified as differentially regulated subsequent to minocycline administration represent exciting targets for future pain studies. Most certainly, a future challenge will be to decipher the exact mechanisms and molecular machinery that give minocycline its sex-specific actions.

## Conflict of interest statement

The authors have no conflicts of interest to declare.

## Appendix A. Supplemental digital content

Supplemental digital content associated with this article can be found online at http://links.lww.com/PAIN/B144.
